# Emergence of MERS-CoV in the Middle East: Origins, Transmission, Treatment, and Perspectives

**DOI:** 10.1371/journal.ppat.1004457

**Published:** 2014-12-04

**Authors:** Ahmad Sharif-Yakan, Souha S. Kanj

**Affiliations:** Department of Internal Medicine-Division of Infectious Diseases, American University of Beirut Medical Center, Beirut, Lebanon; University of Michigan Medical School, United States of America

## Introduction

Two years have passed since the initial description of the Middle East respiratory syndrome coronavirus (MERS-CoV), yet the epidemic is far from being controlled. The high case fatality rate, the recent steep increase in reported cases, and the potential to cause a global pandemic during the upcoming Hajj season are serious concerns. Although a wealth of information about the pathophysiology, proposed animal reservoir, and intermediate host has been revealed, many questions remain unanswered. We herein review MERS-CoV, covering its proposed origins, route of transmission, treatment options, and future perspectives.

## Origin

First reported in 2012 [1], Middle East respiratory syndrome coronavirus (MERS-CoV) is a novel coronavirus and the first lineage 2C *Betacoronavirus* known to infect humans [2]. With a case fatality rate of 35%, an urgent response is needed to prevent a global pandemic [3]. Prior to 2003, coronaviruses were not considered serious human pathogens since they only caused mild upper respiratory tract infections (URTIs) [4]. The first zoonotic introduction of a coronavirus into the human population occurred with the severe acute respiratory syndrome coronavirus (SARS-CoV) in 2002. SARS-CoV caused a global pandemic, with 8,400 recorded cases and 800 deaths [5]. MERS-CoV marks the second known zoonotic introduction of a highly pathogenic coronavirus, probably originating from bats. Three lines of evidence currently support this theory: (1) the very close phylogenetic similarity with the bat *Betacoronaviruses*: BtCoV-HKU4 and BtCoV-HKU5 [6]; (2) closely related coronavirus sequences have been recovered from bats in Africa, Asia, the Americas, and Eurasia; and (3) MERS-CoV uses the evolutionary conserved dipeptidyl peptidase-4 (DPP4) protein in *Pipistrellus pipistrellus* bats for cell entry [7].

Since human-bat contact is limited, camels have been implicated as probable intermediate hosts. MERS-CoV appears to have been circulating in dromedary camels for over 20 years [8]. MERS-CoV uses the DPP4 (CD26) receptor to gain entry and effectively replicate in camel cell lines [9]. Neutralizing antibodies for MERS-CoV have been detected in dromedary camels from Oman, Canary Islands, Egypt, Jordan, United Arab Emirates, and Saudi Arabia [10], [11].

The exact mode of transmission from camels to humans remains to be confirmed [12]. Camel milk was investigated as a possible route of transmission, given the common practice of consuming camel milk in the Arabian Peninsula. The first reported case of MERS-CoV in Yemen occurred in a Yemeni pilot who consumed raw camel milk [13], and Reusken et al. reported the finding of MERS-CoV in camel milk in Qatar [14]. However, respiratory transmission is currently considered as the most likely route of transmission [15]. MERS-CoV has been detected by reverse transcription PCR (RT-PCR) from the nasal swabs of three camels in Qatar and was linked to two confirmed human cases with high similarity upon sequencing, suggesting a possible respiratory mode of transmission [16].

## Human-to-Human Transmission

Several clusters of MERS-CoV cases have been reported, mainly among household members and health care workers (HCWs), suggesting that transmission is through close contact. The largest cluster reported so far has been in 23 HCWs in a hospital in Al Hasa, Kingdom of Saudi Arabia (KSA), while the largest family cluster has been in three infected brothers from Riyadh, KSA [17], [18]. The basic reproductive rate for MERS-CoV has still not been determined with certainty [11]. Using two transmission scenarios, Breban et al. reported an R_0_ of 0.60 and 0.69 [18]. Cauchemez et al. reported a similar R_0_ at 0.63, but warned that in the absence of infection control measures, R_0_ may range from 0.8–1.3 and could lead to a self-sustaining transmission [19]. Propensity for the MERS-CoV to replicate in the lower respiratory tract may account for the observed limited transmission [20]. The United States Centers for Disease Control and Prevention (CDC) recommends standard contact and airborne precautions with the use of an N-95 mask when caring for an infected patient [21].

## Case Definition and Clinical Presentation

The CDC defines a laboratory-confirmed case of MERS-CoV as a patient with a positive PCR from a respiratory sample, and a probable case as a patient who had close contact with a confirmed case but inconclusive laboratory evidence [22]. The incubation period for the presentation of MERS-CoV symptoms is 2–14 days and it remains unknown whether patients are infectious during the incubation period [23]. The average age of presentation is 50 years, with a male predominance [24].

Clinically, MERS-CoV causes symptoms of upper and lower RTIs [23]. The severity of symptoms varies widely. Most asymptomatic cases have been discovered through screening after contact with a known case [2]. Presenting signs and symptoms may include high-grade fever, non-productive cough, dyspnea, headache, myalgia, nausea, vomiting, and diarrhea that may precede the respiratory symptoms [25], [26]. Renal failure has been frequently reported, yet no conclusive evidence of a direct viral invasion of renal tissues exists [11], [27], [28]. Notably, most patients who developed complications had coexisting medical co-morbidities [11]. Laboratory findings on admission may include leukopenia, lymphopenia, thrombocytopenia, and elevated lactate dehydrogenase levels [25]. MERS-CoV can also cause severe pneumonia with acute respiratory distress syndrome (ARDS), requiring mechanical ventilation and intensive care admission [24]. To date, there is still a lack of surgical and pathological information from patients infected with MERS-CoV, which hampers full understanding of the pathogenesis. Lastly, co-infection with other respiratory viruses and with community-acquired bacteria has been also reported in MERS-CoV patients [11], [29], [30].

## Epidemiology

As of June 26, 2014, WHO officially reported 707 affected patients in 21 countries in three continents. Two-hundred and fifty-two patients have died of MERS-CoV, setting the case fatality rate at 35% [3]. The cases so far have been acquired either directly through a probable zoonotic source, or as a result of human–human transmission via close contact. Retrospective analysis tracked the first outbreak to a hospital in the city of Al-Zarqa in Jordan in April 2012 [31]. An unexplained observation has been the seasonal variation in reported numbers, with a peak between April and June of each year. The number of cases reported during April 2014 alone was alarming, because it was greater than the cumulative number of cases reported since the outbreak began [32]. The recent increase in the number of infected patients may arguably be attributed to better case detection and active surveillance programs. Yet other factors may have contributed to the observed surge, including suboptimal infection control practices in affected hospitals in Saudi Arabia, as documented in a recent report of the WHO mission to Jeddah [33]. Another explanation for the seasonal variation may be that it coincides with camel birthing season, and younger camels seem to be more often infected than their older counterparts [34]. The distribution of the total reported cases by country is as follows: 85.8% in the KSA, 8.1% in the United Arab Emirates, 1.7% in Jordan, and 1% in Qatar [32]. Cases have also been reported in Kuwait, Yemen, Oman, Iran, Lebanon, Tunisia, Algeria, Bangladesh, Malaysia, France, Italy, Germany, the Netherlands, United Kingdom, Greece, Italy, and the United States [32]. Furthermore, seropositive camels for MERS-CoV were detected in Egypt, Kenya, Nigeria, Tunisia, and Ethiopia, suggesting that there may be MERS-CoV cases that are undetected in Africa [8], [35], [36].

## A Feared Outbreak: The Hajj Pilgrimage

In 2012, 3,161,573 Muslims from 188 countries gathered in Mecca to perform the annual Hajj, the largest gathering of Muslims in the world. The identification of MERS-CoV in Saudi Arabia has generated international concern of a global pandemic. As a response, the Saudi government requested that elderly and chronically ill Muslims avoid Hajj in 2013 and restricted the number of pilgrims to 2,061,573. Consequently, no cases were reported during the pilgrimage of that year [37]. Nasopharyngeal specimens collected from 5,235 pilgrims revealed no cases of MERS-CoV nasal carriage [38]. A prospective cohort study of 129 French pilgrims did not reveal any MERS-CoV cases [39]. Nevertheless, the potential for spreading of MERS-CoV during the 2014 Hajj season (October 2–6) remains possible, especially since documented transmission occurred this year in patients from Iran and Malaysia after their return from Umrah in Mecca. It is worth noting that the two most frequently visited cities during the Hajj, Mecca and Medina, have so far reported 32 and 35 cases respectively [32].

## Vaccine and Treatment

MERS-CoV binds to the DPP4 (CD26) surface receptor using the spike (S) surface protein with subsequent cell entry [40]. The exact mechanism of entry after receptor binding is still unknown. The S surface protein is composed of a core subdomain that shares similarity with that of SARS-CoV and a receptor binding subdomain (RBSD) that exhibits significant variation from the SARS-CoV RBSD. The development of vaccines targeting the RBSD of MERS-CoV is currently under investigation because they are thought to be safer and more effective than vaccines based on inactivated virus, DNA, or viral vectors [40]. Another potential therapeutic approach is the inhibition of the papain-like and/or 3C-like protease of MERS-CoV [41].

To date, no effective therapy or prophylaxis for MERS-CoV exists. Supportive therapy remains the cornerstone of management. Current treatment is based on previous experience with the SARS-CoV, in-vitro studies, and case series. Various agents have been tried, including those that block virus entry, inhibit viral replication, or interfere with host immune response [42]. The International Severe Acute Respiratory and Emerging Infection Consortium (ISARIC) suggested therapeutic options for treatment of MERS-CoV infection with various agents alongside continuous evaluation of efficacy, and in the setting of clinical trials [43]. Based on experience with SARS-CoV, the use of convalescent plasma, hyper-immune globulin, or human monoclonal antibodies that contain neutralizing antibodies may be efficacious and is recommended as first-line treatment when available [43]. Ribavirin and interferon alpha-2b both showed promising results, especially when used in combination, both in vitro and in animal studies using rhesus macaques monkeys [44]. However, these positive results did not translate clinically in an observational study in five patients, all of whom succumbed to the infection [45], [46]. Repurposing of currently available agents may be an efficient approach. Dyall et al. screened various agents with potential therapeutic efficacy [46]. Cyclosporin A, mycophenolic acid, interferon-beta, homoharringtonine, cycloheximide, anisomycin, and emetine dihydrochloride hydrate were found to have the most potent in vitro activity against MERS-CoV.

## Conclusions and Future Perspectives

Despite the progress in our understanding of MERS-CoV, many questions remain unanswered. The definitive origin, exact mechanism of transmission, and the reason behind seasonal variability are still unclear. Although most cases have been described in countries of the Arabian Peninsula, the increasing travel to the region and the Hajj season in KSA pose a threat of a potential global pandemic. Extensive efforts are required to speed up the development of an effective therapy and vaccine. Repurposing of currently available pharmaceutical agents is highly desirable for a more rapid drug development. Meanwhile, HCWs who encounter patients with respiratory symptoms who have lived or traveled to areas with MERS-CoV should have a low threshold to consider a diagnosis of MERS-CoV, with testing and immediate implementation of proper infection control practices to prevent further spread. Finally, given the important role that camels may play in transmission of the virus, the common practices in the Arabian Peninsula of herding and consuming unpasteurized camels' milk should be discouraged until conclusive evidence is obtained that such practices do not contribute to infection.

## References

[1] Zaki AM, Van Boheemen S, Bestebroer TM, Osterhaus AD, Fouchier RA (2012) Isolation of a novel coronavirus from a man with pneumonia in saudi arabia. N Engl J Med 367: 1814–1820 10.1056/NEJMoa1211721 23075143

[2] Raj VS, Osterhaus AD, Fouchier RA, Haagmans BL (2014) MERS: Emergence of a novel human coronavirus. Curr Opin Virol 5: 58–62 10.1016/j.coviro.2014.01.010 24584035 PMC4028407

[3] World Health Organization (2014) Middle East respiratory syndrome coronavirus (MERS-CoV) summary and literature update as of 26 June 2014. Available: http://www.who.int/csr/don/2014_06_26_mers/en/. Accessed 30 June 2014.

[4] Drosten C, Günther S, Preiser W, Van Der Werf S, Brodt H, et al (2003) Identification of a novel coronavirus in patients with severe acute respiratory syndrome. N Engl J Med 348: 1967–1976 10.1056/NEJMoa030747 12690091

[5] World Health Organization (2003) Consensus document on the epidemiology of severe acute respiratory syndrome (SARS). Available: http://www.who.int/csr/sars/en/WHOconsensus.pdf. Accessed 29 June 2014.

[6] van Boheemen S, de Graaf M, Lauber C, Bestebroer TM, Raj VS, et al (2012) Genomic characterization of a newly discovered coronavirus associated with acute respiratory distress syndrome in humans. MBio 3: e00473–12 10.1128/mBio.00473-12 23170002 PMC3509437

[7] Raj VS, Mou H, Smits SL, Dekkers DH, Müller MA, et al (2013) Dipeptidyl peptidase 4 is a functional receptor for the emerging human coronavirus-EMC. Nature 495: 251–254 10.1038/nature12005 23486063 PMC7095326

[8] Corman VM, Jores J, Meyer B, Younan M, Liljander A, et al (2014) Antibodies against MERS coronavirus in dromedary camels, Kenya, 1992–2013. Emerg Infect Dis 20: 1319–1322 10.3201/eid2008.140596 25075637 PMC4111164

[9] Eckerle I, Corman VM, Muller MA, Lenk M, Ulrich RG, et al (2014) Replicative capacity of MERS coronavirus in livestock cell lines. Emerg Infect Dis 20: 276–279 10.3201/eid2002.131182 24457147 PMC3901466

[10] Reusken CB, Haagmans BL, Müller MA, Gutierrez C, Godeke G, et al (2013) Middle east respiratory syndrome coronavirus neutralising serum antibodies in dromedary camels: A comparative serological study. Lancet Infect Dis 13: 859–866 10.1016/S1473-3099(13)70164-6 23933067 PMC7106530

[11] Milne-Price S, Miazgowicz KL, Munster VJ (2014) The emergence of the Middle East respiratory syndrome coronavirus (MERS-CoV). Pathog Dis 71: 121–136 10.1111/2049-632X.12166 24585737 PMC4106996

[12] Zumla AI, Memish ZA (2014) Middle east respiratory syndrome coronavirus: Epidemic potential or a storm in a teacup? Eur Respir J 43: 1243–1248 10.1183/09031936.00227213 24627533

[13] World Health Organization (2014) Global Alert and Response: Middle East respiratory syndrome coronavirus (MERS-CoV) – update, 07 May 2014. Available: http://www.who.int/csr/don/2014_05_07_mers_yemen/en/. Accessed 30 Aug 2014.

[14] Reusken C, Farag E, Jonges M, Godeke G, El-Sayed A, et al (2014) Middle east respiratory syndrome coronavirus (MERS-CoV) RNA and neutralising antibodies in milk collected according to local customs from dromedary camels, qatar, april 2014. Euro Surveill 19: p20829ii Available: http://www.eurosurveillance.org/ViewArticle.aspx?ArticleId=20829 Accessed 6 November 2014.. 10.2807/1560-7917.es2014.19.23.20829 24957745

[15] Nowotny N, Kolodziejek J (2014) Middle east respiratory syndrome coronavirus (MERS-CoV) in dromedary camels, Oman, 2013. Euro Surveill 19: 15 Available: http://www.eurosurveillance.org/ViewArticle.aspx?ArticleId=20781 Accessed 6 November 2014.. 24786259 10.2807/1560-7917.es2014.19.16.20781

[16] Haagmans BL, Al Dhahiry SH, Reusken CB, Raj VS, Galiano M, et al (2014) Middle east respiratory syndrome coronavirus in dromedary camels: An outbreak investigation. Lancet Infect Dis 14: 140–145 10.1016/S1473-3099(13)70690-X 24355866 PMC7106553

[17] Assiri A, McGeer A, Perl TM, Price CS, Al Rabeeah AA, et al (2013) Hospital outbreak of middle east respiratory syndrome coronavirus. N Engl J Med 369: 407–416 10.1056/NEJMoa1306742 23782161 PMC4029105

[18] Breban R, Riou J, Fontanet A (2013) Interhuman transmissibility of middle east respiratory syndrome coronavirus: Estimation of pandemic risk. Lancet 382: : 694–699.doi:10.1016/S0140-6736(13)61492-0 10.1016/S0140-6736(13)61492-0 PMC7159280 23831141

[19] Cauchemez S, Fraser C, Van Kerkhove MD, Donnelly CA, Riley S, et al. (2014) Middle east respiratory syndrome coronavirus: Quantification of the extent of the epidemic, surveillance biases, and transmissibility. Lancet Infect Dis 14: : 50–56. doi:10.1016/S1473-3099(13)70304-9 10.1016/S1473-3099(13)70304-9 PMC3895322 24239323

[20] de Wit E, Rasmussen AL, Falzarano D, Bushmaker T, Feldmann F, et al (2013) Middle east respiratory syndrome coronavirus (MERS-CoV) causes transient lower respiratory tract infection in rhesus macaques. Proc Natl Acad Sci U S A 110: 16598–16603 10.1073/pnas.1310744110 24062443 PMC3799368

[21] Center for Disease Control and Prevention CDC (2014). Interim infection control and prevention recommendations for hospitalized patients with MERS-CoV. Available: http://www.cdc.gov/coronavirus/mers/infection-prevention-control.html. Accessed 29 June 2014.

[22] Center for Disease Control and Prevention CDC (2014).MERS Case Definition. Available: http://www.cdc.gov/coronavirus/mers/case-def.html. Accessed 29 June 2014.

[23] Infectious Diseases Society of America (2014) MERS clinical update from the IDSA. Available: http://www.magnetmail.net/actions/email_web_version.cfm?recipient_id=124961864&message_id=4470910&user_id=IDSociety&group_id=1191715&jobid=18802511. Accessed 29 June 2014.

[24] Center for Disease Control and Prevention CDC (2014) MERS clinical Features. Available: http://www.cdc.gov/coronavirus/mers/clinical-features.html. Accessed 29 June 2014.

[25] Assiri A, Al-Tawfiq JA, Al-Rabeeah AA, Al-Rabiah FA, Al-Hajjar S, et al (2013) Epidemiological, demographic, and clinical characteristics of 47 cases of middle east respiratory syndrome coronavirus disease from saudi arabia: A descriptive study. Lancet Infect Dis 13: 752–761 10.1016/S1473-3099(13)70204-4 23891402 PMC7185445

[26] Guery B, Poissy J, el Mansouf L, Séjourné C, Ettahar N, et al. (2013) Clinical features and viral diagnosis of two cases of infection with middle east respiratory syndrome coronavirus: A report of nosocomial transmission. Lancet 381: : 2265–2272. doi:10.1016/S0140-6736(13)60982-4 10.1016/S0140-6736(13)60982-4 PMC7159298 23727167

[27] Memish ZA, Zumla AI, Al-Hakeem RF, Al-Rabeeah AA, Stephens GM (2013) Family cluster of middle east respiratory syndrome coronavirus infections. N Engl J Med 368: 2487–2494 10.1056/NEJMoa1303729 23718156

[28] Guberina H, Witzke O, Timm J, Dittmer U, Müller M, et al (2014) A patient with severe respiratory failure caused by novel human coronavirus. Infection 42: 203–206 10.1007/s15010-013-0509-9 23900771 PMC7099911

[29] Omrani AS, Matin MA, Haddad Q, Al-Nakhli D, Memish ZA, et al (2013) A family cluster of middle east respiratory syndrome coronavirus infections related to a likely unrecognized asymptomatic or mild case. Int J Infect Dis 17: e668–e672 10.1016/j.ijid.2013.07.001 23916548 PMC7110537

[30] Drosten C, Seilmaier M, Corman VM, Hartmann W, Scheible G, et al. (2013) Clinical features and virological analysis of a case of middle east respiratory syndrome coronavirus infection. Lancet Infect Dis 13: : 745–751. doi:10.1016/S1473-3099(13)70154-3 10.1016/S1473-3099(13)70154-3 PMC7164791 23782859

[31] Hijawi B, Abdallat M, Sayaydeh A, Alqasrawi S, Haddadin A, et al (2013) Novel coronavirus infections in jordan, april 2012: Epidemiological findings from a retrospective investigation. Emhj 19: 1. 23888790

[32] MERS Corona map. Available: http://coronamap.com. Accessed 29 June 2014

[33] World Health Organization (2014) WHO concludes MERS-CoV mission in Saudi Arabia, May 2014. Available: http://www.emro.who.int/media/news/mers-cov-mission-saudi-arabia.html. Accessed 9 August 2014.

[34] Holmes D (2014) MERS-CoV enigma deepens as reported cases surge. The Lancet 383: 1793 10.1016/S0140-6736(14)60866-7 PMC7137153 24868566

[35] Reusken CB, Messadi L, Feyisa A, Ularamu H, Godeke GJ, et al.. (2014) Geographic distribution of MERS coronavirus among dromedary camels, africa. Emerging Infect Dis 20: 1370–1374. doi:10.3201/eid2008.140590 10.3201/eid2008.140590 PMC4111168 25062254

[36] Chu DK, Poon LL, Gomaa MM, Shehata MM, Perera R, et al (2014) MERS coronaviruses in dromedary camels, egypt. Emerg Infect Dis 20: 1049–1053 doi 10.3201/eid2006.140299. 24856660 PMC4036765

[37] World Health Organization (2014) Middle East respiratory syndrome coronavirus (MERS-CoV) summary and literature update as of 20 Jan 2014. Available: http://www.who.int/csr/disease/coronavirus_infections/MERS_CoV_Update_20_Jan_2014.pdf. Accessed 29 June 2014.

[38] Memish ZA, Assiri A, Almasri M, Alhakeem RF, Turkestani A, et al. (2014) Prevalence of MERS-CoV nasal carriage and compliance with the saudi health recommendations among pilgrims attending the 2013 hajj. J Infect Dis 210: : 1067–1072. doi:10.1093/infdis/jiu150 10.1093/infdis/jiu150 PMC7107361 24620019

[39] Gautret P, Charrel R, Benkouiten S, Belhouchat K, Nougairede A, et al. (2014) Lack of MERS coronavirus but prevalence of influenza virus in french pilgrims after 2013 hajj. Emerg Infect Dis 20: : 728–730. doi:10.3201/eid2004.131708 10.3201/eid2004.131708 PMC3966380 24656283

[40] Zhou N, Zhang Y, Zhang J, Feng L, Bao J (2014) The receptor binding domain of MERS-CoV: The dawn of vaccine and treatment development. J Formosan Med Assoc 113: 143–147 10.1016/j.jfma.2013.11.006 24342026 PMC7127315

[41] Yang X, Chen X, Bian G, Tu J, Xing Y, et al (2014) Proteolytic processing, deubiquitinase and interferon antagonist activities of middle east respiratory syndrome coronavirus papain-like protease. J Gen Virol 95: 614–626 10.1099/vir.0.059014-0 24362959

[42] Al-Tawfiq JA, Memish ZA (2014) What are our pharmacotherapeutic options for MERS-CoV? Expert Rev Clin Pharmacol 7: 235–238 10.1586/17512433.2014.890515 24580083

[43] Brown C, Carson G, Chand M, Zambon M (2014) International Severe Acute Respiratory & Emerging Infection Consortium (ISARIC)- Clinical Decision Making Tool for Treatment of MERS-CoV v.1.1. Available: http://www.hpa.org.uk/webc/HPAwebFile/HPAweb_C/1317139281416. Accessed 29 June 2014.

[44] Yao Y, Bao L, Deng W, Xu L, Li F, et al (2014) An animal model of MERS produced by infection of rhesus macaques with MERS coronavirus. J Infect Dis 209: 236–242 10.1093/infdis/jit590 24218506 PMC7107340

[45] Al-Tawfiq JA, Momattin H, Dib J, Memish ZA (2014) Ribavirin and interferon therapy in patients infected with the middle east respiratory syndrome coronavirus: An observational study. Int J Infect Dis 20: 42–46 10.1016/j.ijid.2013.12.003 24406736 PMC7110882

[46] Dyall J, Coleman CM, Hart BJ, Venkataraman T, Holbrook MR, et al (2014) Repurposing of clinically developed drugs for treatment of middle east respiratory coronavirus infection. Antimicrob Agents Chemother 58: 4885–4893 10.1128/AAC.03036-14 24841273 PMC4136000

